# MRI-Guided Salvage Focal Cryoablation: A 10-Year Single-Center Experience in 114 Patients with Localized Recurrent Prostate Cancer

**DOI:** 10.3390/cancers15164093

**Published:** 2023-08-14

**Authors:** Yvonne Wimper, Christiaan G. Overduin, J. P. Michiel Sedelaar, Jeroen Veltman, Sjoerd F. M. Jenniskens, Joyce G. R. Bomers, Jurgen J. Fütterer

**Affiliations:** 1 Department of Medical Imaging, Radboud Institute for Health Sciences, Radboud University Medical Center, 6525 GA Nijmegen, The Netherlands; kristian.overduin@radboudumc.nl (C.G.O.); sjoerd.jenniskens@radboudumc.nl (S.F.M.J.); joyce.bomers@radboudmc.nl (J.G.R.B.); jurgen.futterer@radboudumc.nl (J.J.F.); 2 Department of Urology, Radboud Institute for Health Sciences, Radboud University Medical Center, 6525 GA Nijmegen, The Netherlands; michiel.sedelaar@radboudumc.nl; 3 Department of Radiology, Ziekenhuisgroep Twente, 7609 PP Almelo, The Netherlands; j.veltman@zgt.nl

**Keywords:** prostatic neoplasms, magnetic resonance imaging (MRI)-guided, focal therapy, focal salvage cryoablation, localized prostate cancer, recurrence, adult, male

## Abstract

**Simple Summary:**

Patients with localized recurrent prostate cancer (PCa) are typically treated with hormonal therapy, surgery (radical prostatectomy), or radiation therapy. These treatments are associated with serious side-effects such as incontinence and impotence. Magnetic resonance imaging (MRI)-guided focal cryoablation is an emerging treatment for this patient group. This therapy aims to treat the cancer selectively while preserving the rest of the prostatic gland and thereby minimizing the risk of treatment toxicity. Cryoablation promises to delay and potentially avoid the initiation of other salvage treatments. In this article, we investigate the ability to achieve disease control and to avoid the need for other salvage treatments afterwards. Our research provides long-term evidence on oncological and safety outcomes in a large group of patients treated with MRI-guided focal cryoablation. We also investigated the influence of prior treatment on patient outcomes.

**Abstract:**

Patients with localized recurrent prostate cancer (PCa) are eligible for androgen-deprivation therapy, salvage radical prostatectomy (RP) or radiation therapy. These treatments are associated with serious side-effects, illustrating the need for alternative local treatment options with lower morbidity rates. All patients who underwent magnetic resonance imaging (MRI)-guided salvage focal cryoablation (SFC) with localized recurrent PCa between 2011–2021 (*n* = 114) were included. Two subgroups were formed: patients without (*n* = 99) and with prior RP (*n* = 15). We assessed the recurrence- (RFS) and treatment-free survival (TFS), measured from date of treatment to date of recurrence or initiation of additional salvage treatment, using Kaplan–Meier plots. Complications were reported using the Clavien–Dindo (CD) scale. Overall 1-year and 5-year RFS were 76.0% and 25.1%, and overall 1-year and 5-year TFS were 91.5% and 58.2%, respectively. Patients without prior RP showed a significantly higher 1-year (78.5% vs. 52.5%) and 5-year RFS (28.1% vs. 0.0%; *p* = 0.03), and a trend towards a higher 1-year (92.6% vs. 79.0%) and 5-year TFS (60.2% vs. 23.0%; *p* = 0.10) compared to those with prior RP. A total of 46 complications occurred in 37 patients, and the overall complication rate was 32.4% (37/114 patients). The majority (41/46; 89.1%) of complications were minor (CD 1–2). Overall (31.3 vs. 40.0%) and major (3.0 vs. 13.3%) complication rates were lower in patients without compared to those with prior RP, respectively. MRI-guided SFC is an effective and safe therapy for patients with recurrent PCa, and has proved to delay and potentially prevent the initiation of salvage treatments. Patients with locally recurrent PCa after prior RP had an increased risk of recurrence, a shortened time to additional treatment, and more complications compared to those without prior RP, which should be considered when selecting patients for SFC.

## 1. Introduction

Patients diagnosed with recurrent prostate cancer (PCa) have different whole-gland treatment options depending on their primary treatment. Salvage radiation therapy (RT) may provide a cure for patients with localized recurrence after radical prostatectomy (RP), and additional androgen-deprivation therapy (ADT) can be offered to patients with a biochemical recurrence (BCR) [1,2]. However, ADT has a considerable impact on quality of life, with side-effects including depression, cardiovascular disease, osteoporosis, sexual dysfunction and fatigue [3,4,5]. Moreover, despite the initial high response rate to ADT, 10–20% of the patients develop castration-resistant PCa within five years, and 33% develop metastases within two years [6]. In cases of histologically proven local recurrence after RT, highly selected patients can be treated with salvage RP. Salvage RP lacks the systemic treatment-related toxicity of ADT, yet it is associated with an increased risk of urinary incontinence (22–90% at 1-year) and a decreased erectile function rate (0–13.1%) [7].

Ultrasound-guided salvage focal cryoablation (SFC) has emerged as an alternative treatment that seems to offer comparable oncologic outcomes to whole-gland salvage standard therapies, but with lower morbidity rates [8,9]. Ultrasound-guided cryoablation is recognized as an established treatment option for men with newly diagnosed or radiorecurrent organ-confined PCa [1,10].

Magnetic resonance imaging (MRI)-guidance of cryoablation offers several benefits over ultrasound, pertaining to its capability of high-contrast visualization of the tumor and iceball, multiplanar needle guidance, near real-time volumetric monitoring of the frozen tissue extent, and indirect information about the achieved temperature [11,12,13]. Thereby, treatment may be more accurately monitored and tailored to the specific tumor location, enabling the concept of focal treatment. To date, there is little evidence on MRI-guided cryoablation in prostate cancer, and previous studies are inhomogeneous in their patient selection (native [14,15,16] vs. recurrent PCa after RP [17] or RT [14,16,18,19,20]) and in the type of ablation (focal [14,17,18,19,20] vs. whole-gland cryoablation [15,16]). Previously, we have described the initial experience and short-term follow-up of MRI-guided SFC in localized radio-recurrent PCa [18]. The aim of the present study is to evaluate the long-term oncological and safety outcomes of MRI-guided SFC of localized recurrent PCa in a 10 year single-center cohort. In addition, we investigated the influence of prior treatment on patient outcomes.

## 2. Materials and Methods

### 2.1. Patient Selection and Characteristics

**Figure 1:**
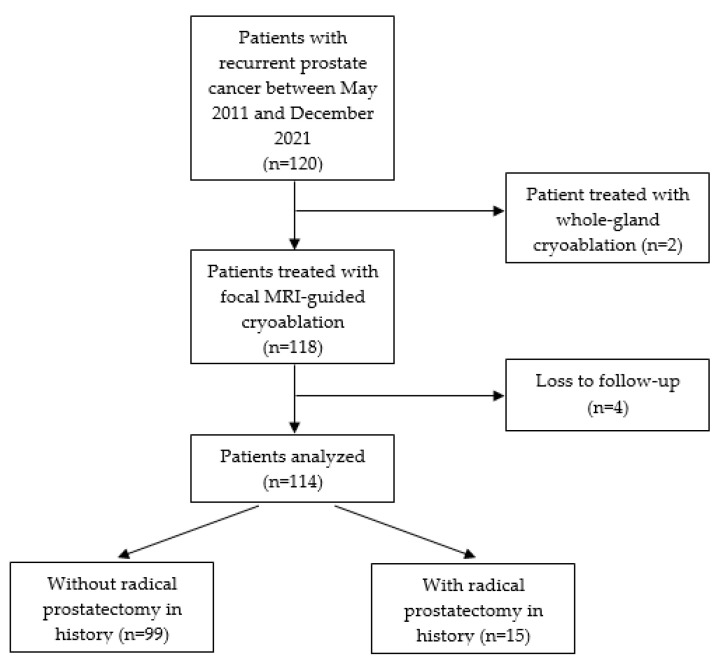
Retrospective cohort study design and flow chart.

After approval from the local institutional review board, all patients who underwent percutaneous MRI-guided SFC for localized recurrent PCa from May 2011 to December 2021 were retrospectively identified at least 12 months after treatment. The inclusion criteria were patients treated with MRI-guided SFC for recurrent localized PCa. A total number of 118 patients met the inclusion criteria (Figure 1). Four patients were lost to follow-up and therefore excluded from analysis. A total of 114 patients were included for final analysis, and data were collected from their electronic medical records and retrospectively analyzed.

All patients were diagnosed with localized recurrent PCa based on a pelvic multiparametric MRI (mpMRI) and/or prostate-specific membrane antigen (PSMA) positron emission tomography (PET)/computed tomography (CT). Subsequent in-bore MRI-guided prostate biopsy was performed for histopathological confirmation. Metastatic screening was performed using the abdominal MRI protocol and, as of 2017, a PSMA PET/CT. Patients were eligible for SFC in case of negative metastatic screening and a localized recurrent PCa that was deemed locally treatable with focal cryoablation. In cases wherein cryoablation was deemed unsafe or wherein we expected an inability to achieve complete tumor coverage, e.g., extensive tumor size, macroscopic rectal or bladder wall invasion, or broad contact with the rectal wall without the ability to obtain adequate spacing, patients were not offered focal cryoablation. The decision to perform MRI-guided SFC was made by the multidisciplinary tumor board.

### 2.2. Treatment Procedures and Follow-Up

**Figure 2:**
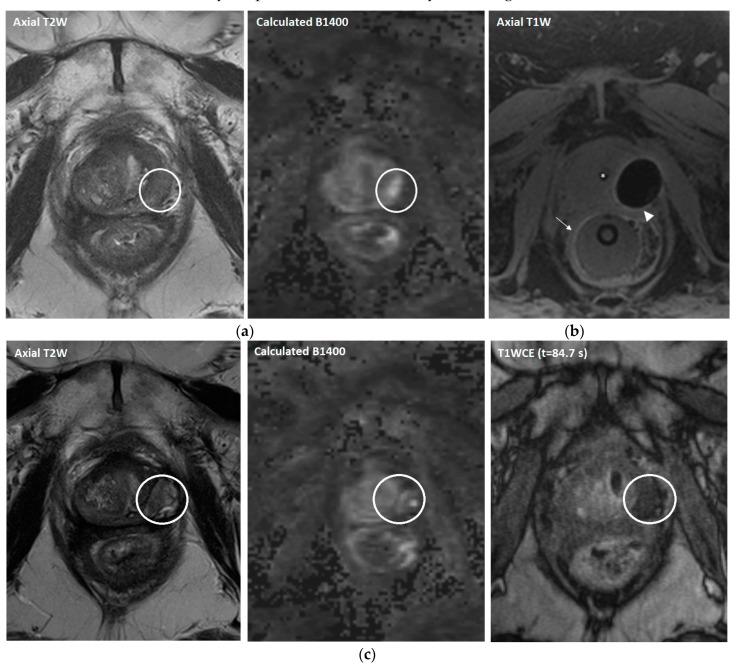
(**a**) Pre-treatment axial T2W and high-*b*-value MR images in a 61-year-old patient with recurrent prostate cancer in the peripheral zone of the left mid gland (circle) with a PSA level of 2.0 ng/mL (Gleason score 4 + 4 = 8) after initial treatment with external beam radiation therapy. (**b**) The axial T1W MR image demonstrates the cryoablation under near real-time guidance with two cryoneedles (ice-seeds). The urethra (*) and rectum (arrow) contain a urethral warming catheter and a rectal balloon, respectively. The iceball is sharply visualized as a signal void area surrounded by a hyperintense rim corresponding to 0 °C (arrowhead). (**c**) Post-treatment axial T2W, high-*b*-value, and contrast-enhanced MR images at 3 months follow-up after cryoablation (PSA level of 1.2 ng/mL) showing no evidence of residual disease or recurrence (circle).

Prior to the cryoablation procedure, antibiotic prophylaxis was prescribed (ciprofloxacin 500 mg twice daily for 5 days), starting one day before the procedure according to hospital guidelines. Patients were treated under general anesthesia in a lithotomy position either in a 1.5 or 3 Tesla MR system (MAGNETOM Avanto or Skyra, Siemens Healthineers, Erlangen, Germany). Cryoablation was performed with an MR-compatible cryoablation device (MRI-SeedNet; Galil Medical, Yokneam, Israel). A urethral warming catheter and rectal balloon were inserted in order to prevent damage to the urethra and rectal wall (Figure 2). MR-compatible cryoneedles (IceRod and IceSeed; Galil Medical, Yokneam, Israel) were placed transperineally under real-time MRI guidance, after which two 10-min freezing cycles were applied followed by 2 min of passive and 1 min of active thawing. During cryoablation, near real-time MR imaging was performed in order to continuously evaluate iceball growth and tumor coverage, as well as the adjacent structures. After the procedure, a transurethral catheter was inserted for one night in order to prevent acute urinary retention. Additional details on the treatment protocol have been previously described [19,20].

Follow-up consisted of urology visits at 1, 3, 6, 9, and 12 months after treatment, and included prostate-specific antigen (PSA) measurement and a check for complications. In addition, patients routinely underwent mpMRI at 3, 6, and 12 months. At 12 months’ follow-up, patients were offered a targeted MRI-guided biopsy from the ablation zone to evaluate the success of the treatment. Additional targeted biopsies were also taken in case of suspicion of recurrent or residual disease outside the treatment zone on imaging. After the first year, patients were followed by the urologist in a 3- to 6-month intervals.

### 2.3. Outcomes

The primary outcomes were recurrence-free (RFS) and treatment-free survival (TFS) and postprocedural complications, reported according to the Clavien–Dindo (CD) System for Reporting Complications [21]. The RFS and TFS were measured from the date of cryoablation to the date of any recurrence or date of initiation of additional salvage treatment other than cryoablation (i.e., salvage RP, ADT, or chemotherapy) due to recurrent PCa, respectively. We defined any recurrence (both local and metastatic) in the case of a radiological, a histopathological recurrence, or a BCR, according to the Phoenix definition (PSA > nadir + 2 ng/mL) [22].

The secondary outcomes were biochemical disease-free survival (BDFS), measured as the time between cryoablation and BCR, and overall survival (OS), defined as the time between cryoablation and the patients’ death. Subgroup analysis was performed for patients with and without prior history of RP as a potential risk factor for complications after cryoablation.

### 2.4. Statistical Analysis

Statistical analyses were performed using SPSS Statistics (version 27). A paired *t*-test was used for assessing the change in PSA level compared to baseline. Differences between the two subgroups in outcomes were determined by using the Chi-square test for categorical variables. Survival outcomes were assessed using the Kaplan–Meier method. A log-rank analysis was used to assess differences between survival curves. Finally, a Cox proportional hazard analysis was used to determine the association between survival outcomes and prior history of RP. Two-sided *p*-values ≤ 0.05 were considered statistically significant.

## 3. Results

### 3.1. Patient, Tumor and Treatment Characteristics

A total of 114 men (median age 68 years; range 52–83) who underwent MRI-guided SFC met the inclusion criteria. A total of 114 procedures were performed with a mean procedure time of 124.5 min. The mean hospitalization duration was 1.2 days. The International Society of Urological Pathology (ISUP) Grade Groups before cryoablation are shown in Table 1. In a total of 22 patients, the ISUP grade groups were not available, either due to confirmed recurrence based on positive PSMA PET/CT and BCR, or due to signs of malignancy in the biopsy without the possibility of the pathologist determining a definitive Gleason score, due to their history of RT. Detailed baseline patient and tumor characteristics are summarized in Table 1.

### 3.2. Oncological Outcomes

#### 3.2.1. Recurrence- and Treatment-Free Survival

The mean follow-up time was 58.6 months. Overall, 78/114 (68.4%) patients had a recurrence after a mean time of 28.3 months. Local recurrence occurred in most patients (*n* = 42 [36.8%]), 8 (7.0%) of whom were classified as having residual disease, with a mean time to local recurrence of 33.1 months. Moreover, a total of 16/114 (14.0%) had a lymph node metastasis. A total of 20/114 (17.5%) patients developed distant metastases and only 5 (4.4%) patients had a distant metastasis after we implemented PSMA PET/CT in the patient selection procedure in 2017. Seventeen (14.9%) patients had bone metastasis, one (0.9%) patient had lung metastases, and two (1.8%) patients had both bone and lung metastases (Supplementary Materials Table S1). Overall RFS and TFS rates were 76.0% and 91.5% at 1 year vs. 25.1% and 58.2 at 5 years, respectively (Table 2). A total of 47/114 (41.2%) required the initiation of additional salvage treatment after a mean time of 34.0 months, with most patients requiring at least additional ADT (39/47 [83.0%]; Supplementary Materials Table S2).

**Figure 3:**
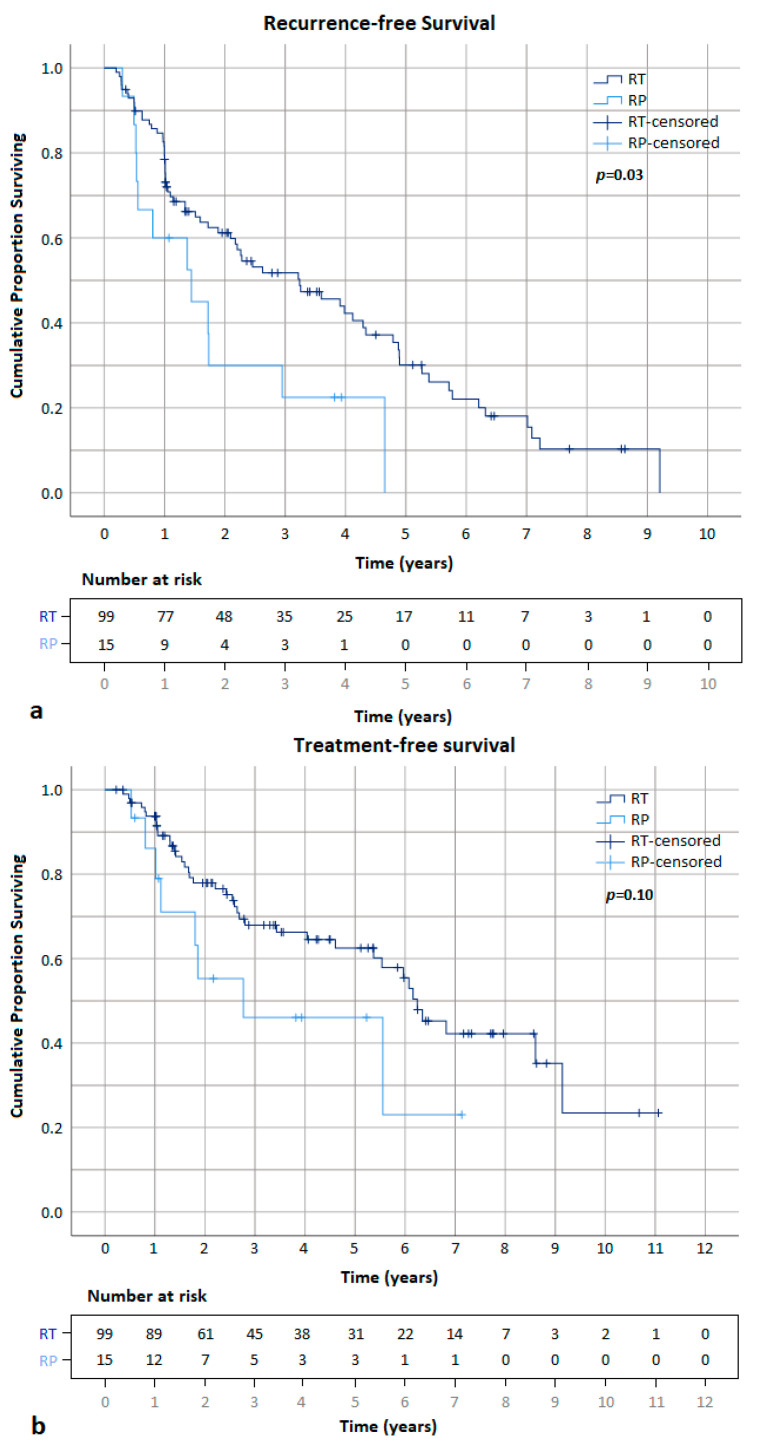
Recurrence- (**a**) and treatment-free (**b**) survival curves between the two subgroups without (*n* = 99) and with (*n* = 15) prior history of RP.

When comparing the two subgroups, fewer patients (34/99 [34.3%]) had a local recurrence without prior RP compared to with prior RP (8/15 [53.3%]) after a mean time of 32.5 and 11.6, respectively. A Kaplan–Meier analysis demonstrates a significantly higher RFS and a trend towards a higher TFS in patients without vs. with prior RP, with a 1-year and 5-year RFS of 78.5% and 28.1% (*p* = 0.03) and a 1-year and 5-year TFS of 92.6% and 60.2% (*p* = 0.10; Figure 3). On univariate analysis, patients with prior RP had a significantly higher probability of developing recurrence (HR 2.02; *p* = 0.03) and an insignificantly increased likelihood of requiring salvage treatment (HR 1.89; *p* = 0.11; Table 2).

#### 3.2.2. Biochemical Disease-Free and Overall Survival

Overall BDFS and OS rates were 81.4% and 98.9% at 1 year vs. 36.6% and 90.8 at 5 years, respectively (Table 2). Divided between the subgroups, patients without prior RP had a higher 1-year and 5-year BDFS (82.6% and 40.2%) compared to those with prior RP (65.2% vs. 0.0%; *p* = 0.04).

#### 3.2.3. Post-Treatment Follow-up

**Figure 4:**
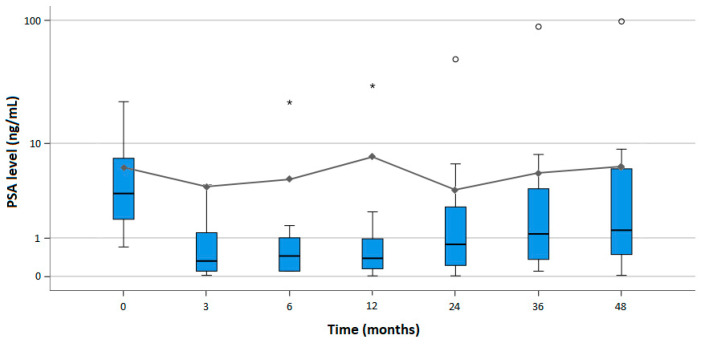
Box plots representing the observed change in serum prostate-specific antigen (PSA) levels (ng/mL) before and after cryoablation (*n* = 114), including an overlaying line plot representing the mean, over the time in months (* and ° showing outliers and extreme outliers, respectively).

Overall, PSA levels showed a significant reduction at 3 and 24 months [3.47 and 3.22 ng/mL; *p* < 0.001 and *p* = 0.04, respectively], and an insignificant reduction at 6 and 36 months [4.20 and 4.86 ng/mL; *p* = 0.06 and *p* = 0.95, respectively], as compared to the mean baseline level of 5.59 ng/mL (Supplementary Materials Table S3). At 12 and 48 months’ follow-up, the PSA levels were insignificantly higher [7.25 and 5.67 ng/mL; *p* = 0.59 and *p* = 0.86, respectively] compared to baseline. A detailed distribution of the changes in serum PSA levels (ng/mL) is shown in Figure 4.

The majority of patients (68 [59.6%]) did not undergo a 12-month targeted biopsy due to confirmed metastases based on imaging or BCR, or due to low PSA levels combined with a negative mpMRI result. A total of 46 out of 144 patients (40.4%) received a 12-months biopsy. Out of the 11 patients with a positive biopsy, 2 (18.2%) and 9 (81.8%) patients had a negative and positive 12-month MRI scan, respectively. Furthermore, out of the 35 patients with a negative biopsy, 34 (97.1%) and 1 (2.9%) patients had a negative and positive 12-month MRI scan, respectively.

### 3.3. Procedural Safety and Complications

Of a total of 114 procedures, 1 (0.9%) intraprocedural complication occurred. During insertion of the urethral warming catheter, a faux route was created, which was noted on MRI. The patient received a transurethral catheter, and treatment was postponed for 8 weeks.

Post-procedurally, a total of 46 complications were recorded in 37/114 (32.5%) patients. Of these, one complication occurred in 28 patients and two complications in the remaining 9 patients. The majority of complications (41/46 [89.1%]) were limited to CD grade 1–2. The major complication rate (CD grade ≥ 3) was 4.4% (5/114 patients). The most common complication was acute urinary retention (*n* = 18 [15.8%]), followed by lower urinary tract symptoms/incontinence (*n* = 7 [6.1%]) and rectourethral fistula (*n* = 6 [5.3%]; Supplementary Materials Table S4). Fistula formation occurred after a mean of 235 days after cryoablation. Three (2.6%) patients were treated conservatively, two (1.8%) patients underwent a pelvic exenteration, and one (0.9%) patient underwent a cystoprostatectomy. One patient (0.9%) suffered from erectile dysfunction. One grade 4a complication has been reported. A patient with an extensive cardiovascular history was diagnosed with an acute myocardial infarction one day post-cryoablation. He underwent a percutaneous coronary intervention and was administered for surveillance to the coronary care unit for one week due to a cardiogenic shock. A detailed description of the complications after cryoablation is provided in Table 3.

When comparing the subgroups, 31/99 (31.3%) patients without history of RP reported ≥ 1 complication, as compared to 6/15 (40.0%) patients with prior RP. Major complications were seen in 3/99 (3.0%) and in 2/15 (13.3%) patients without and with RP in history, respectively. Although the highest incidence of complications was seen in patients with prior RP, this difference was statistically insignificant (odds ratio 1.46, 95% confidence interval 0.48–4.47; *p* = 0.50).

## 4. Discussion

Over recent years, salvage focal cryoablation has been established as a local treatment alternative to systemic treatment in patients with localized PCa recurrence. Two recent systematic reviews have demonstrated treatment outcomes in local salvage therapies for localized non-metastatic radiorecurrent PCa. They reported 5-year BDFS rates between 44–65% in salvage ultrasound-guided cryoablation in radiorecurrent PCa [8,23], which appears to be in line with the 5-year BDFS of 40.2% and 5-year TFS of 58.2% in our cohort. The five-year RFS, on the other hand, was 28.1% in our cohort. The RFS used in this study has a more strict definition of recurrence, combining biochemical, radiological and histopathological findings, as we deemed this to be more appropriate to the focal therapy setting. The commonly used Phoenix definition for biochemical failure was originally validated for recurrence after external beam radiation therapy [22], and in focal therapy, unlike in radical whole-gland treatment, the non-treated part of the prostate gland is still able to produce PSA. When comparing the 5-year BDFS to that of other local salvage treatments in radio-recurrent PCa, the outcomes were the following: 49–59% in RP, 61–71% (3-year BDFS) in focal brachytherapy, 52–67% in high-dose-rate brachytherapy (HDR), 48–63% in low-dose-rate brachytherapy (LDR), 43–63% in high-intensity focused ultrasound (HIFU) and 60% in stereotactic body radiotherapy (SBRT) [8,23]. In our cohort, a total of 78/114 (68.4%) patients had a recurrence after cryoablation, most of whom had a local recurrence (*n* = 42 [36.8%]). Furthermore, the overall mean time to recurrence and time to local recurrence were 28.3 and 33.1 months, respectively. Although to date, there is little evidence on long-term oncological outcomes in MRI-guided cryoablation, one study reported a comparable mean time to local progression of 775 days (≈25.8 months) [16]. However, this study differs from our cohort, since its authors performed whole-gland MRI-guided cryoablation in patients with native and radio-recurrent PCa. Considering this time to recurrence and the fact that in focal therapy, unlike in whole-gland radical treatment, there is a higher likelihood of recurrence in the remaining prostatic tissue, we would recommend a strict protocol including a PSA test, mpMRI, and prostate biopsy. More data are needed on the optimal follow-up protocol.

A total of 20/114 (17.5%) men developed distant metastasis after MRI-guided SFC. The majority of these occurred before the introduction of PSMA PET/CT to our routine pre-treatment work-up, where metastatic screening still consisted of pelvic bone and lymph node MRI. In recent years, PSMA PET/CT has shown superior accuracy for the detection of pelvic nodal disease and distant metastases compared to the combined findings of CT and bone scans [24]. After the introduction of PSMA PET/CT since 2017, distant metastasis occurred in only 5 (4.4%) patients during follow-up.

A systematic review on functional outcomes after local salvage therapies for radiorecurrent PCa showed incontinence rates varying between 48–72%, 0–13% and 0–40% following salvage RP, focal and whole-gland cryoablation, respectively [25]. Our cohort had relatively low incontinence rates of 6.1%. As for the development of fistulas, in ultrasound-guided cryoablation, the rates ranged from 0–4.7% (follow-up between 30 days–53 months) compared to a total incidence of 5.3% in our study (follow-up 58.6 months) [25,26]. However, only three (2.6%) patients had a major fistula requiring active treatment. The majority of fistulas occurred during the first two years after the implementation of cryoablation in our hospital, and may be contributed to a learning curve, i.e., proper patient selection and balancing iceball growth between radical treatment and avoiding complications. Moreover, we treated a large proportion of patients with a tumor in the peripheral zone (59.6%) and in the seminal vesicles (17.5%); these patients are more at risk for developing rectourethral fistula.

Our study shows that patients with a history of RP have worse oncological outcomes than patients without prior RP. Most local recurrences after RP, e.g., at the level of the anastomosis, are not suitable for SFC. The majority of the patients in the RP group had a recurrence in the seminal vesicles. Since this patient group had tumors in more advanced stages, this might have contributed to their increased risk of recurrence and shortened time to additional treatment. Furthermore, patients treated with prior RP had more major complications compared to patients without prior RP treatment. This may be due to the fact that we treated a relatively small number of patients in the RP group, thereby limiting our learning curve in balancing iceball growth in this patient group. Lastly, patients with RP in history were more at risk of developing a recurrence and complications, possibly due to the close relationship between the tumor and the adjacent structures (i.e., rectum and urethra), resulting in a small ablation zone.

This study is limited by its retrospective design and the limited number of patients in the post-prostatectomy cohort. We also lacked objective follow-up data on validated questionnaires for measuring rates for incontinence and erectile function. The main strength of this work is that it provides long-term results on one of the largest cohorts treated with MRI-guided SFC for recurrent PCa. Our 10-year follow-up shows that MRI-guided SFC is a suitable technique for treating local recurrent PCa, which can delay and in some cases prevent other salvage treatments with minimal complications. The future perspective of MRI-guided SFC will need to be shaped by achieving a consensus on appropriate patient selection, the optimal sequence of alternative therapies, such as ADT of radioimmunotherapy, and a proper follow-up protocol to enable adequate long-term management of localized PCa recurrence. Future research concerning rectal wall displacement with saline [27,28], absorbable hydrogel rectal spacers (SpaceOAR) [29], and transperineal injection of autologous blood [30] could also prove whether these techniques are beneficial for increasing the safety margins of the iceball to the rectal wall, and thereby reducing the risk of developing rectovesical fistula.

## 5. Conclusions

MRI-guided salvage focal cryoablation is an effective and safe therapy for patients with localized recurrent prostate cancer, and has proved to delay and potentially prevent the initiation of salvage treatments. During patient selection, it should be considered that patients treated with prior radical prostatectomy have an increased risk of recurrence, a shortened time to additional treatment, and the risk major complications compared to those without prior radical prostatectomy treatment.

## Figures and Tables

**Table 1:** Baseline patient, tumor, and treatment characteristics.

Patient Characteristics	Overall (*n* = 114)		
Prior RP ^1^ treatment, *n* (%)			
No	99 (86.8)		
EBRT ^2^	68 (59.6)		
Brachytherapy	28 (24.6)		
EBRT + brachytherapy	3 (2.6)		
Yes	15 (13.2)		
RP only	3 (2.6)		
RP + EBRT	12 (10.5)		
**Patient characteristics**	Overall (*n* = 114)	Without RP (*n* = 99)	With RP (*n* = 15)
Age at cryoablation (years), median (IQR) ^3^	68 (64–72)	68 (64–72)	66 (64–70)
Prior ADT ^4^, *n* (%)			
Yes	44 (38.6)	39 (39.4)	5 (33.3)
No	70 (61.4)	60 (60.6)	10 (66.7)
PSA ^5^ level before initial treatment (ng/mL), median (IQR)	12.8 (7.7–19.9)	12.5 (7.7–19.2)	15.5 (8.8–25.5)
PSA level at baseline (ng/mL), median (IQR)	3.9 (2.4–6.8)	4.2 (2.7–7.4)	2.5 (1.2–5.9)
**Tumor characteristics**	Overall (*n* = 114)	Without RP (*n* = 99)	With RP (*n* = 15)
Tumor localization, *n* (%)			
Peripheral zone	68 (59.6)	64 (64.6)	4 (26.7)
Transition zone	26 (22.8)	23 (23.2)	3 (20.0)
Seminal vesicles	20 (17.5)	12 (12.1)	8 (53.3)
ISUP ^6^ Grade group at baseline, *n* (%)			
1	7 (6.1)	7 (7.1)	0 (0.0)
2	24 (21.1)	22 (22.2)	2 (13.3)
3	19 (16.7)	15 (15.2)	4 (26.7)
4	20 (17.5)	18 (18.2)	2 (13.3)
5	22 (19.3)	20 (20.2)	2 (13.3)
NA ^7^	22 (19.3)	17 (17.2)	5 (33.3)

^1^ RP = radical prostatectomy. ^2^ EBRT = external beam radiation therapy. ^3^ IQR = interquartile range. ^4^ ADT = androgen-deprivation therapy. ^5^ PSA = prostate-specific antigen. ^6^ ISUP = International Society of Urological Pathology Grade Groups. ^7^ NA = not available.

**Table 2:** Univariable and multivariable analysis for detecting predictors of recurrence-free, treatment-free and overall survival in patients with recurrent prostate cancer (*n* = 114).

	1-Year, % (95% CI) ^1^	5-Year, % (95% CI)	*p* Value	HR ^2^ (95% CI)	*p* Value
**Recurrence-free survival**					
Overall	76.0 (68.0–84.0)	25.1 (14.9–35.3)	–	–	–
Prior RP ^3^ treatment			0.03	2.02 (1.07–3.80)	0.03
No	78.5 (70.1–86.9)	28.1 (11.2–39.3)			
Yes	52.5 (26.3–78.7)	0.0 (0.0–0.0)			
**Biochemical disease-free survival**					
Overall	81.4 (74.0–88.8)	36.6 (25.4–47.8)	–	–	–
Prior RP treatment			0.04	2.07 (1.04–4.15)	0.04
No	82.6 (75.0–90.2)	40.2 (28.0–52.4)			
Yes	65.2 (39.8–90.6)	0.0 (0.0–0.0)			
**Treatment-free survival**					
Overall	91.5 (86.3–96.7)	58.2 (47.0–69.4)	–	–	–
Prior RP treatment			0.10	1.89 (0.87–4.07)	0.11
No	92.6 (87.2–98.0)	60.2 (48.2–72.2)			
Yes	79.0 (57.4–100.0)	23.0 (0.0–58.6)			
**Overall survival**	98.9 (96.8–100.0)	90.8 (83.6–98.0)	–	–	–

^1^ CI = confidence interval. ^2^ HR = hazard ratio. ^3^ RP = radical prostatectomy.

**Table 3:** Complications after cryoablation in 37 patients according to the Clavien–Dindo System for Reporting Complications, subdivided into patients without (*n* = 31) and with prior RP ^1^ (*n* = 6).

	Overall Complications	Complications without Prior RP Treatment	Complications with Prior RP Treatment
Grade 1, *n* (%)	40 (87.0)	35 (89.7)	5 (71.4)
Grade 2, *n* (%)	1 (2.2)	1 (2.6)	0 (0.0)
Grade 3a, *n* (%)	0 (0.0)	0 (0.0)	0 (0.0)
Grade 3b, *n* (%)	4 (8.7)	2 (5.1)	2 (28.6)
Grade 4a, *n* (%)	1 (2.2)	1 (2.6)	0 (0.0)
Grade 4b, *n* (%)	0 (0.0)	0 (0.0)	0 (0.0)
Grade 5, *n* (%)	0 (0.0)	0 (0.0)	0 (0.0)
Overall, *n* (%)	46 (100.0)	39 (100.0)	7 (100.0)

^1^ RP = radical prostatectomy.

## Data Availability

The data presented in this article are available on request from the corresponding author.

## Supplementary Materials

The following supporting information can be downloaded at: https://www.mdpi.com/article/10.3390/cancers15164093/s1, Table S1: Recurrence characteristics after MRI-guided focal salvage cryoablation; Table S2: Overview of the different types of additional salvage treatments after MRI-guided focal salvage cryoablation (*n* = 114); Table S3: Mean PSA level (ng/mL) changes after cryoablation compared to baseline over the time in months; Table S4: Complications after cryoablation in 37 patients according to the Clavien–Dindo System for Reporting complications subdivided by type in a total cohort of 114 patients.
