# Supplementary material for: MRI-Guided Salvage Focal Cryoablation: A 10-Year Single-Center Experience in 114 Patients with Localized Recurrent Prostate Cancer
Source: Cancers (Basel). 2023 Aug 14;15(16):4093. doi: 10.3390/cancers15164093 (PMC10452144; doi:10.3390/cancers15164093)
Supplement: Supplementary file 1 [file cancers-15-04093-s001.zip › cancers-2504857-supplementary.pdf]

Table S1. Recurrence characteristics after MRI-guided focal salvage cryoablation.

Type of Recurrence	n (%)		
	**Overall (*n* = 114)**	**Without RP [1] (*n* = 99)**	**With RP (*n* = 15)**
Local recurrence	42 (36.8)	34 (34.3)	8 (53.3)
Based on imaging	25 (21.9)	19 (19.2)	6 (40.0)
Based on histopathological verification	17 (14.9)	15 (15.2)	2 (13.3)
Lymph node metastasis	16 (14.0)	14 (14.1)	2 (13.3)
Based on imaging	13 (11.4)	11 (11.1)	2 (13.3)
Based on histopathological verification	3 (2.6)	3 (3.0)	0 (0.0)
Distant metastasis based on imaging	20 (17.5)	18 (18.2)	2 (13.3)
Bone metastasis	17 (14.9)	15 (15.2)	2 (13.3)
Lung metastasis	1 (0.9)	1 (1.0)	NA [2]
Both lung and bone metastases	2 (1.8)	2 (2.0)	NA
Overall	78 (68.4)	66 (66.7)	12 (80.0)

[1] RP = radical prostatectomy. [2] NA = not applicable.

Table S2. Overview of the different types of additional salvage treatments after MRI-guided focal salvage cryoablation (*n* = 114).

Treatment Type	n (%)
No salvage treatment	67 (58.8)
ADT [1] only	27 (23.7)
Salvage prostatectomy only	3 (2.6)
ADT + salvage prostatectomy	5 (4.4)
ADT + chemotherapy	5 (4.4)
ADT + salvage prostatectomy + chemotherapy	2 (1.8)
Others	5 (4.4)

[1] ADT = androgen-deprivation therapy.

Table S3. Mean PSA [1] level (ng/mL) changes after cryoablation compared to baseline over the time in months.

Time (Months)	Overall (*n* = 114)	*p* Value	Without RP [2] (*n* = 99)	*p* Value	With RP (*n* = 15)	*p* Value
0	5.59	–	5.75	–	4.54	–
3	3.47	<0.001	3.58	<0.001	2.84	0.18
6	4.20	0.06	4.06	0.04	5.05	0.74
12	7.25	0.59	6.16	0.74	14.02	0.22
24	3.22	0.04	2.59	<0.001	7.69	0.56
36	4.86	0.95	3.24	0.13	16.77	0.49
48	5.67	0.86	3.47	0.07	24.88	0.53

[1] PSA = prostate-specific antigen. [2] RP = radical prostatectomy.

Table S4. Complications after cryoablation in 37 patients according to Clavien-Dindo System for Reporting Complications subdivided by type in a total cohort of 114 patients.

Type Complications	n (%)	Time to Complication in Days after Cryoablation Mean (Median)
AUR [1]	18 (15.8)	3 (2)
LUTS [2]/incontinence	7 (6.1)	29 (28)
Rectourethral fistula	6 (5.3)	235 (112)
Hematuria	5 (4.4)	23 (18)
UTI [3]	4 (3.5)	20 (22)
Erectile dysfunction	1 (0.9)	43 (NA [4])
Other	5 (4.4)	13 1)
Overall	46 (40.4)	43 (8)

[1] AUR = acute urinary retention. [2] LUTS = lower urinary tract symptoms. [3] UTI = urinary tract infection. [4] NA = not applicable.
